# Is There a Role for HTLV-1-Specific CTL in Adult T-Cell Leukemia/Lymphoma?

**DOI:** 10.1155/2012/391953

**Published:** 2011-11-30

**Authors:** Aileen G. Rowan, Charles R. M. Bangham

**Affiliations:** Department of Immunology, Wright-Fleming Institute, Imperial College London, London W2 1PG, UK

## Abstract

ATLL is an aggressive malignancy of T cells that affects about 5% of individuals infected with HTLV-1. The precise mechanism of oncogenesis is not known, but there is evidence that two regulatory viral proteins, Tax and HBZ, are involved. A high set point proviral load is associated with development of ATLL or a chronic inflammatory condition, HAM/TSP. Several lines of evidence, including HLA class 1 association studies and in vitro killing assays, indicate that cytotoxic T lymphocytes are instrumental in determining this proviral load set point. Prior studies have focused chiefly on the CTL response to the immunodominant Tax protein: efficient lysis of Tax-expressing cells inversely correlates with proviral load in nonmalignant infection. However, a recent study showed that strong binding of peptides from HBZ, but not Tax, to HLA class 1 molecules was associated with a low proviral load and a reduced risk of developing HAM/TSP, indicating an important role for HBZ-specific CTL in determining infection outcome. In comparison with nonmalignant infection, HTLV-1-specific CTLs in ATLL patients are reduced in frequency and functionally deficient. Here we discuss the nature of protective CTL responses in nonmalignant HTLV-1 infection and explore the potential of CTLs to protect against ATLL.

## 1. Introduction

Human T-cell lymphotropic virus-1 (HTLV-1) is a retrovirus which predominantly infects CD4^+^ T cells, where it is reverse transcribed and integrates into host DNA. The integrated provirus can then disseminate by de novo infection of T cells via the virological synapse, or by inducing clonal expansion of the host cell. Most infected individuals do not experience any symptoms, and HTLV-1-associated disease is rarely observed in individuals with a proviral load of less than 1% of their peripheral blood mononuclear cells (PBMCs) [1]. Approximately 2–6% of individuals HTLV-1 develop adult T-cell leukemia/lymphoma (ATLL), and a slightly lower percentage suffer from inflammatory disorders, including HTLV-1-associated myelopathy/tropical spastic paraparesis (HAM/TSP). ATLL is a highly aggressive T-cell malignancy with a poor prognosis, and, even with standard treatment, the median survival time for clinically acute forms of the disease is measured in months [2, 3]. Chemotherapeutic intervention has had limited efficacy despite the development of drugs to specifically target ATLL cells, though some improvement has been reported combining antiviral drugs (zidovudine) and immunomodulators (such as type-1 interferon) [3–5]. Allogeneic hematopoietic stem cell transplantation (HSCT) has also improved survival [6], though may not be a viable treatment option in all cases due to the advanced age of most ATLL patients and the lack of suitable donors [2]. Thus, the ability to harness the host immune system to control ATLL would be an attractive proposition. 

Leukemic cells carry at least one copy of the provirus, and express CD4, the IL-2 receptor alpha chain CD25, and typically exhibit a flower-like multilobed nucleus and genetic abnormalities. Downregulation of CD3 and CD7 on leukemic cells has been described [7], and several groups have detected FoxP3 protein in ATLL cells [7–9], although the degree of expression of FoxP3 varies between patients. There is conflicting evidence on whether FoxP3-expressing leukemic cells have regulatory capacity [9, 10]. ATLL is also characterized by uncontrolled expansion of T cells which share a common T-cell receptor-V*β* chain, implying that the disease is a result of malignant transformation and expansion of a small number of infected cells. High-throughput quantitative sequencing of the site of proviral integration reveals that the bulk of the proviral load in ATLL patients is composed of highly abundant clones with a small number of unique proviral integration sites. This contrasts with asymptomatic carriers (ACs) of the virus and patients with HAM/TSP, whose infected cells consist of a mixture of T-cell clones carrying many unique integration sites with varying abundance in the peripheral blood [11]. 

CD8^+^ cytotoxic T lymphocyte (CTL) recognition of viral peptides presented in the context of the human leukocyte antigen (HLA) class 1 (major histocompatibility (MHC) protein class 1) triggers cytokine production by the CTL and lysis of the infected cell. The affinity and stability of the interaction between a given TCR and peptide-MHC complex must exceed a threshold level to allow initial activation of the T cell and to induce clonal expansion to form effector and memory T cells. The nature of a CTL response to its cognate antigen can subsequently be modulated by cell-intrinsic and cell-extrinsic factors, which include the concentration of peptide-MHC complexes on the target cell surface, the length of time which has passed since the CTL has encountered antigen, expression of the peptide-MHC-TCR-stabilizing CD8 co-receptor, inhibition by regulatory T cells, and the cytokine microenvironment. Thus, the efficiency of CTL control is determined by a combination of the host genotype and environmental factors. 

HTLV-1 antigens are expressed in vivo and induce a CTL response. In particular, the viral transcription factor Tax is widely recognized by CTLs, and abundant activated Tax-specific CTL can be detected in most infected individuals [12–15]. Tax is expressed early in the life cycle of the virus (ex vivo data) and can induce proliferation of infected cells, but also expression of other viral genes, and contains several well-characterized CTL epitopes. CTL epitopes and CTLs specific for other viral genes have been described; though Tax is immunodominant [12, 16], efficient recognition of other subdominant target antigens appears to be more important in protection: see what follows. 

 Little is known about the role of HTLV-1-specific CTL in preventing or resolving ATLL. There is anecdotal evidence that immune control of ATLL is achievable: spontaneous regression of ATLL is sometimes observed. One report documents such a patient, in which regression coincided with acquisition of an ability of their PBMCs to kill an ATLL cell line in vitro. Subsequent disease progression was accompanied by a decline in the ability of their PBMC to lyse ATLL cells [17]. Furthermore, there have been several reports of ATLL emergence in HTLV-1^+^ individuals after immune suppressive treatment during liver [18] and renal [19] transplantation, indicating the importance of continuous immune surveillance in infected individuals in suppression of ATLL development. 

In this paper, we review

- what is known about the HTLV-1-specific CTL response in ATLL patients;
- properties of HTLV-1 specific CTLs in patients who have a low proviral load;
- the role of HLA class 1 genes in CTL control of HTLV-1;
- expression of CTL target antigens by ATLL cells.

## 2. HTLV-1-Specific CTL Response in ATLL Patients

Few data have been published describing the functional properties of CTLs in ATLL, but all reports agree that the ex vivo CTL response to HTLV-1 antigens is weak in these patients [20]. Using a panel of peptide-MHC tetramers Kozako et al. found that, in comparison with asymptomatic individuals, Tax-specific CTLs in ATLL patients were directed at a narrower range of epitopes and present at lower frequencies or were undetectable ex vivo [21]. Env-specific CTLs were also detected but were present at low frequencies and only in asymptomatic carriers. Expression of the programmed death-1 (PD-1) receptor, which delivers inhibitory signals to the CTL, was slightly higher on virus-specific CTLs in ATLL patients than in ACs [22]. A subset of ATLL patients have no detectable CTL when PBMCs are assayed directly ex vivo. After culture, however, functional CTL are sometimes observed [23, 24]: efficient killing of ATLL cells can be demonstrated by CD8^+^ T cells after being cultured in vitro for a week in the presence of IL-2 and a polyclonal activator or stimulated with HLA-matched infected cells [23, 24]. 

In peripheral blood of acute ATLL patients, the lymphocyte population consists predominantly of leukemic CD4^+^CD25^+^ cells. Careful analysis revealed that although the ratio of CD4^+^: CD8^+^ T cells is grossly elevated, the absolute number of CD8^+^ T cells detected in peripheral blood is similar to the normal range or reduced by a factor of two [20, 23]. Thus, depletion of the CD8^+^ T-cell subset does not explain the reduced CTL activity observed. A more likely candidate mechanism to explain this phenomenon is suppression by regulatory T cells. Uninfected FoxP3^+^ T cells with regulatory activity are significantly expanded in ATLL patients, and lysis of autologous-infected cells by uncultured CTLs was inversely proportional to the frequency of FoxP3^+^ cells [9]. It is also possible that competition from ATLL cells for resources such as growth factors and cytokines, for example, IL-2, disrupts the antiviral CTL response.

Despite the observed deficiency in the CTL response to HTLV-1 in ATLL, there is evidence for an efficient immune response in a subset of ATLL patients. In three individuals with ATLL, Furukawa et al. observed an amino acid change within the CTL epitope Tax [11–19] which rendered Tax unrecognizable to CTLs specific for the consensus epitope [25]. In addition, premature stop codons within the *tax* gene which abrogate its transactivating activity were observed in another ATLL patient. Familial analysis revealed that the mutation emerged after viral transmission [25], strongly implying that immune selection favoured the variant genes. Loss of Tax expression or activity has the downstream effect of reduced expression of other viral genes such as *gag*, *pol,* and *env* which are transactivated by Tax. Thus, by ablating a single viral protein, the infected cell may also escape surveillance by CTL specific for other viral proteins. This *tax* mutation was not restricted to ATLL patients, but was also detectable in a minor population of infected cells in ACs and patients with HAM/TSP [26]. There is further evidence of large deletions within the HTLV-1 genome in dominant ATLL clones. In particular, the 5′-long terminal repeat (LTR) region, which encodes the promoter that drives expression of the *tax* gene, is deleted in 28% of ATLL patients, compared with 4% ACs [25, 27, 28]. It is less clear whether these large deletions are due to immune selection or to an inherent genetic instability of the leukemic cells (see Table 1).

## 3. CTL Control of HTLV-1 Proviral Load

Analysis of HTLV-1-specific CTL responses in individuals who do not develop disease may provide important clues as to the character of a desirable CTL response in ATLL. In cases of nonmalignant infection, proviral load can vary by up to 1000-fold between individuals, though within an individual the load usually remains stable over years of infection. In addition, ACs with a viral load above 1% are significantly more likely to develop inflammatory disease [1, 29]. Thus, we identify a successful immune response as one that results in a low proviral load set point. Many methods have been employed to quantify the frequency of HTLV-1-specific CTLs: primarily identifying cells by functional assays or directly staining reactive TCRs using fluorochrome-labelled peptide-MHC tetramers. However, simply measuring the frequency of HTLV-1-specific CTLs has revealed a positive, negative, or zero correlation with proviral load in different studies [30–32]. This is not an unexpected finding in persistent infection [33], where chronic antigen exposure can drive proliferation of CTLs which are not protective and may even be detrimental in the case of HTLV-1-associated inflammatory disorders. Thus, the absolute frequency of HTLV-1-specific CTLs may not be informative, and alternative methods of quantification of CTL efficiency are desirable.

We have developed a method to quantify the efficiency with which uncultured CD8^+^ cells suppress Tax expression in autologous infected CD4^+^ T cells in a perforin-dependent and MHC class-I-restricted manner. CD8^+^-depleted PBMCs are mixed with autologous CD8^+^ T cells at a range of effector: target ratios and cocultured overnight, following which the number of Tax-expressing cells is enumerated by flow cytometry, and the rate of elimination of Tax-expressing cells is estimated by using nonlinear regression to fit a mathematical model [34]. Using this assay, we have found that the rate of elimination of Tax-expressing cells is inversely proportional to proviral load, suggesting that a more efficient CTL response has the effect of lowering proviral load [34]. The CTL efficiency parameter measured by this assay could explain up to 50% of the observed variation among infected individuals in proviral load [34]. Although the precise epitope specificity of CTLs responsible for elimination of Tax-expressing cells is not defined in this assay, we also observed that the rate of infected cell elimination is proportional to the sensitivity with which Tax-specific CTLs detect peptides presented on target cells and that naturally infected cells expressing high levels of Tax protein are killed significantly faster than cells expressing lower levels of Tax [35]. These observations indicate that Tax-specific CTLs can eliminate autologous cells which begin to express the provirus. This is not just a phenomenon observed in cells cultured in vitro: in vivo, CD4^+^CD45^+^RO cells (which comprise most Tax-expressing cells) proliferate faster in infected individuals than in uninfected individuals and tend to die faster in HTLV-1-infected people [36]. Taken with the observation that, in ACs and patients with HAM/TSP, proviral load is relatively stable over years of infection [11], these data are consistent with the existence of a dynamic equilibrium between proviral expression and CTL surveillance, in which the per-cell efficiency of CTLs is a major determinant of the proviral load set point.

## 4. HLA Class 1 and the Immune Response to HTLV-1

Some of the most powerful and compelling evidence for the important role of CTLs in controlling HTLV-1 comes from genetic studies. In 1999, Jeffery et al. provided the first definitive evidence that HLA class 1 genes can be protective in HTLV-1 infection [37]. They observed that asymptomatic individuals who possessed HLA-A*02 alleles had a proviral load which was approximately one third that of individuals who lacked HLA-A*02. In addition, the chance of developing HAM/TSP was halved in HLA-A*02^+^ individuals. HLA-A* 02 is a common and highly polymorphic allele, present at high frequencies in most populations. HLA-A*0201 binds the Tax 11–19 epitope with extremely high affinity, and up to 10% of circulating CTLs can be specific for this epitope [38]. In ATLL, Yashiki et al. reported that the HLA class 1 alleles HLA-A*26, HLA-B*4002, HLA-B*4006, and HLA-B*4801 were more abundant in individuals who develop ATLL in comparison with AC [39]. They hypothesize that this is due to a reduced ability to bind and present Tax peptides, due to the inability of these alleles to bind anchoring residues in peptides derived from Tax [39].

Although Tax is both expressed and frequently targeted by CTL in HTLV-1 infection, a recent genetic study has shifted the focus of attention to another viral protein: HTLV-1 basic leucine zipper protein (HBZ) [40]. HLA association studies are hampered by the heterogeneity of HLA class 1 alleles in a population. Thus, for rare alleles that may be protective, large numbers of participants are required in a given study for associations to reach statistical significance, and the results may only apply to certain populations. Also, association studies do not reveal the precise mechanism of protection these alleles confer. As an alternative approach, MacNamara et al. conducted a systematic study that tested the capacity of an individual's HLA alleles to bind HTLV-1 peptides and asked whether this metric correlated with the proviral load and the “risk” (i.e., relative prevalence) of HAM/TSP. They used experimentally validated epitope prediction software [41] which predicted the affinity of binding of peptides derived from the HTLV-1 genome to HLA-A and -B alleles in a cohort of 202 ACs and 230 patients with HAM/TSP [42]. They found that the known protective alleles A*0201 and C*0801 bound peptides from HBZ with significantly higher affinity than alleles which were associated with disease progression (B*5401). Further analysis showed that in general ACs had HLA alleles which bound peptides from HBZ significantly more strongly than patients with HAM/TSP, and this was not attributable simply to A*0201, C*0801, and B*5401. The more alleles an individual possessed that strongly bound peptides from HBZ, the lower their proviral load. Interestingly, despite the strong protective effect of effective HBZ presentation, peptides from HBZ bound to class 1 molecules with significantly lower affinity than peptides from Tax. These data were unexpected but robust, and although CTL specific for HBZ could only be detected at low frequencies in 25–40% of infected individuals [42, 43], a CTL clone specific for HBZ could efficiently kill HLA-matched primary HTLV-1-infected CD4^+^CD25^+^ cells, indicating that peptides derived from HBZ are presented by naturally infected cells [42].

## 5. Expression of CTL Target Antigens by ATLL Cells

Viral antigen expression by ATLL cells in vivo is of course the critical determinant of their susceptibility to lysis by HTLV-1-specific CTL (see Table 1). Although the Tax protein is strongly expressed in cultured cells of ~50% of ATLL cases [44], Tax mRNA is typically undetectable or present at extremely low copy number in peripheral blood [45, 46]. Despite this, ATLL cells which were immediately fixed ex vivo were sufficient to induce a Tax-specific CTL response when used to immunize rats [44]. These data suggest that CTLs are more sensitive than antibody staining in detecting protein expression and lack of detection of mature protein expression does not preclude the presentation of peptides in vivo. A rat model of ATLL in which HTLV-1-transformed cell lines are adoptively transferred into an immunodeficient host has generated data which supports a role of Tax-specific CTL response in prophylaxis against disease development. Immunization with a DNA vaccine encoding full-length Tax protein or with Tax oligopeptides protects against development of a lymphoproliferative disorder in this model [47, 48]. Similarly, adoptive transfer of Tax-specific CTLs was also sufficient to prevent disease [47]. Interestingly, epitopes from Tax are also presented and targeted by newly expanded CTLs in vivo in ATLL patients who have undergone successful HSCT [49, 50]. 

HBZ is emerging as an alternative viral target antigen in ATLL. Uniquely among HTLV-1 transcripts, several variably spliced isoforms of HBZ mRNA are readily detectable in vivo in all HTLV-1^+^ individuals tested [51]. The negative strand of the 3′ genomic region of HTLV-1, from which HBZ is transcribed, is uniformly conserved in all ATLL cases [28, 52], implying an essential role of HBZ in ATLL persistence, but also making it an attractive target for immunotherapy. The ratio of HBZ mRNA to Tax mRNA is significantly higher in ATLL patients than in patients with HAM/TSP or ACs [51] which may be accounted for by the silencing of Tax by mutation, deletion, or methylation of the Tax-encoding DNA frequently observed in ATLL. Like Tax, however, expression of HBZ protein is usually undetectable in HTLV-1^+^ PBMCs directly ex vivo. Currently available antibodies reveal extremely low expression of HBZ in cultured, naturally infected cells [53]. Evidence from several groups suggests that HBZ protein is likely to be expressed at lower levels than Tax [53] (Rowan, Bangham, unpublished observations), and the majority of the HBZ mRNA is retained in the nucleus [54], which may inhibit its translation. Suemori et al. performed an elegant study which tested the activity of an in vitro generated CTL clone specific for HBZ 24–36 in the context of A*0201. They showed that sensitive, efficient CTLs specific for HBZ could be generated, at least in A*0201^+^ individuals, despite the low binding affinity of HBZ peptides for HLA class 1. These cells could lyse HLA-matched HBZ-transfected or peptide-loaded cells, but not freshly isolated ATLL cells in vitro [53]. More testing is needed to ascertain the contribution of HBZ-specific CTLs in controlling proviral load in vivo, and the rat model of leukemogenesis provides one possible system in which to address this question.

## 6. Discussion

It is clear that the CTL response is crucial in maintaining a low proviral load in nonmalignant cases of HTLV-1 infection. It is also evident that the CTL response to HTLV-1 proteins in ATLL is somewhat lacking, with a reduced frequency of HTLV-1-specific CTLs, a reduced number of viral epitopes targeted, and CTL functional defects. Enhancing weak CTL responses could be a potentially effective, specific method for controlling ATLL: the epitopes are expressed only by infected T cells, and efficient HTLV-1-specific CTLs can provide surveillance for the lifetime of the host; this could be achieved by therapeutic vaccination. In addition, induction or enhancement of HTLV-1-specific CTLs in people at risk of developing ATLL could prevent disease development. Key considerations in designing a vaccine include the choice of antigen, method of delivery, and the need to surmount whatever mechanisms result in a weak HTLV-1-specific CTL response in individuals who develop ATLL. Tax and HBZ are emerging as the key target antigens to be included in a CTL-directed vaccine. Interestingly, both Tax and HBZ have been implicated in the initiation of leukemogenesis, reviewed in [55, 56], and inclusion in a putative vaccine would require functional inactivation of both of these proteins. In fact, several vaccine constructs designed to induce Tax-specific CTLs have already been successfully tested in animal models [57, 58]. HBZ, though less well characterized, plays an indispensable role in maintenance of ATLL cells [52], and the region of the HTLV-1 genome which encodes HBZ appears to be essential as it is preferentially protected from deletion. Since HBZ is constitutively expressed in vivo and ranks as the most protective CTL target antigen in nonmalignant infection, targeting HBZ could be the most important component of a vaccine, particularly for ATLL. It seems logical to target both proteins in order to exploit both the antigenicity of Tax and the potentially highly protective properties of HBZ-specific CTLs. Targeting epitopes from other HTLV-1 proteins may also be beneficial in ATLL; however, no experimental data has been published which supports or indeed refutes this. Finally, it may be advantageous to consider combining therapeutic vaccination with chemotherapeutic ablation of ATLL cells, to increase the effective CTL: target ratio, enabling the CTLs to regain control of the malignancy.

## Figures and Tables

**Table 1:** HBZ and Tax as CTL target antigens: genetics, expression, and antigenicity in ATLL.

	Tax	HBZ
*Naturally occurring genetic and epigenetic changes in ATLL*		

Point mutations	Detected in Tax 11–19 A*0201 epitope [25]	None described

Deletions	Premature stop codons [26]	None described
	Deletions of 5′end of genome [25, 27, 28]	

Epigenetic silencing	Hypermethylation of 5′LTR [46, 59, 60] and pX region [59]	None described

*Expression in ATLL*		

In vivo	mRNA and protein low/undetectable [45, 46]	Multiply spliced isoforms of mRNA detected [51, 52]

Ex vivo cultures	Increased mRNA transcription in vitro culture [54]	Increased mRNA transcription after culture in vitro [54]
	Protein detected in 50% of ATL cases [44]	Protein at threshold for detection using currently available antibodies [53]

*CTL response in AC and HAM/TSP*		

CTL response in vivo	High frequencies Tax-specific CTL detected in 66–94% infected individuals [12, 15, 43]	Low frequencies of HBZ-specific CTL detectable in 25–40% of individuals [42, 43]

Immunogenicity for CTL	Immunodominant, HTLV-1 protein most frequently recognized by CTL [12–14]	Peptides bind weakly to HLA class-1 in general [42] Highly sensitive, cytotoxic CTL which recognise HBZ 26–34 in the context of A*0201 can be generated in vitro [53]

Potential for protection in ATLL	Enhanced ability to present Tax peptides does not confer a significant protective effect [42] but can lyse ATL cells expressing Tax in vitro [23, 24]	Efficient presentation of HBZ peptides significantly associated with low PVL and remaining asymptomatic [42]

## References

[1] Nagai M, Usuku K, Matsumoto W (1998). Analysis of HTLV-I proviral load in 202 HAM/TSP patients and 243 asymptomatic HTLV-I carriers: high proviral load strongly predisposes to HAM/TSP. *Journal of NeuroVirology*.

[2] Shimoyama M (1991). Diagnostic criteria and classification of clinical subtypes of adult T-cell leukaemia-lymphoma. A report from the lymphoma study group (1984–1987). *The British Journal of Haematology*.

[3] Bazarbachi A, Plumelle Y, Carlos Ramos J (2010). Meta-analysis on the use of zidovudine and interferon-alfa in adult T-cell leukemia/lymphoma showing improved survival in the leukemic subtypes. *Journal of Clinical Oncology*.

[4] Gill PS, Harrington W, Kaplan MH (1995). Treatment of adult T-cell leukemia-lymphoma with a combination of interferon alfa and zidovudine. *The New England Journal of Medicine*.

[5] Hermine O, Bouscary D, Gessain A (1995). Brief report: treatment of adult T-cell leukemia-lymphoma with zidovudine and interferon alfa. *The New England Journal of Medicine*.

[6] Kami M, Hamaki T, Miyakoshi S (2003). Allogeneic haematopoietic stem cell transplantation for the treatment of adult T-cell leukaemia/lymphoma. *The British Journal of Haematology*.

[7] Tian Y, Kobayashi S, Ohno N (2011). Leukemic T cells are specifically enriched in a unique CD3^dim^CD7^low^ subpopulation of CD4^+^ T cells in acute-type adult T-cell leukemia. *Cancer Science*.

[8] Abe M, Uchihashi K, Kazuto T (2008). Foxp3 expression on normal and leukemic CD4^+^CD25^+^ T cells implicated in human T-cell leukemia virus type-1 is inconsistent with Treg cells. *European Journal of Haematology*.

[9] Toulza F, Nosaka K, Takiguchi M (2009). FoxP3+ regulatory T cells are distinct from leukemia cells in HTLV-1-associated adult T-cell leukemia. *International Journal of Cancer*.

[10] Chen S, Ishii N, Ine S (2006). Regulatory T cell-like activity of Foxp3+ adult T cell leukemia cells. *International Immunology*.

[11] Gillet NA, Malani N, Melamed A (2011). The host genomic environment of the provirus determines the abundance of HTLV-1-infected T-cell clones. *Blood*.

[12] Goon PKC, Biancardi A, Fast N (2004). Human T Cell Lymphotropic virus (HTLV) type-1-specific CD8^+^ T cells: frequency and immunodominance hierarchy. *Journal of Infectious Diseases*.

[13] Jacobson S, Shida H, McFarlin DE, Fauci AS, Koenig S (1990). Circulating CD8^+^ cytotoxic T lymphocytes specific for HTLV-I pX in patients with HTLV-I associated neurological disease. *Nature*.

[14] Kannagi M, Harada S, Maruyama I (1991). Predominant recognition of human T cell leukemia virus type I (HTLV-I) pX gene products by human CD8^+^ cytotoxic T cells directed against HTLV-I-infected cells. *International Immunology*.

[15] Parker CE, Daenke S, Nightingale S, Bangham CRM (1992). Activated, HTLV-1-specific cytotoxic T-lymphocytes are found in healthy seropositives as well as in patients with tropical spastic paraparesis. *Virology*.

[16] Pique C, Connan F, Levilain JP, Choppin J, Dokhélar MC (1996). Among all human T-cell leukemia virus type 1 proteins, tax, polymerase, and envelope proteins are predicted as preferential targets for the HLA-A2- restricted cytotoxic T-cell response. *Journal of Virology*.

[17] Jinnohara T, Tsujisaki M, Sasaki S, Hinoda Y, Imai K (1997). Cytotoxic activity in a case of adult T-cell leukemia/lymphoma with spontaneous regression. *International Journal of Hematology*.

[18] Suzuki S, Uozumi K, Maeda M (2006). Adult T-cell leukemia in a liver transplant recipient that did not progress after onset of graft rejection. *International Journal of Hematology*.

[19] Hoshida Y, Li T, Dong Z (2001). Lymphoproliferative disorders in renal transplant patients in Japan. *International Journal of Cancer*.

[20] Shimizu Y, Takamori A, Utsunomiya A (2009). Impaired tax-specific t-cell responses with insufficient control of HTLV-1 in a subgroup of individuals at asymptomatic and smoldering stages. *Cancer Science*.

[21] Kozako T, Arima N, Toji S (2006). Reduced frequency, diversity, and function of human T cell leukemia virus type 1-specific CD8^+^ T cell in adult T cell leukemia patients. *Journal of Immunology*.

[22] Kozako T, Yoshimitsu M, Fujiwara H (2009). PD-1/PD-L1 expression in human T-cell leukemia virus type 1 carriers and adult T-cell leukemia/lymphoma patients. *Leukemia*.

[23] Arnulf B, Thorel M, Poirot Y (2004). Loss of the ex *vivo* but not the reinducible CD8^+^ T-cell response to Tax in human T-cell leukemia virus type 1-infected patients with adult T-cell leukemia/lymphoma. *Leukemia*.

[24] Kannagi M, Sugamura K, Kinoshita KI (1984). Specific cytolysis of fresh tumor cells by an autologous killer T cell line derived from an adult T cell leukemia/lymphoma patient. *Journal of Immunology*.

[25] Furukawa Y, Kubota R, Tara M, Izumo S, Osame M (2001). Existence of escape mutant in HTLV-I tax during the development of adult T-cell leukemia. *Blood*.

[26] Furukawa Y, Tara M, Izumo S, Arimura K, Osame M (2006). HTLV-I viral escape and host genetic changes in the development of adult T cell leukemia. *International Journal of Cancer*.

[27] Tamiya S, Matsuoka M, Etoh KI (1996). Two types of defective human T-lymphotropic virus type I provirus in adult T-cell leukemia. *Blood*.

[28] Miyazaki M, Yasunaga JI, Taniguchi Y, Tamiya S, Nakahata T, Matsuoka M (2007). Preferential selection of human T-cell leukemia virus type 1 provirus lacking the 5' long terminal repeat during oncogenesis. *Journal of Virology*.

[29] Okayama A, Stuver S, Matsuoka M (2004). Role of HTLV-1 proviral DNA load and clonality in the development of adult T-cell leukemia/lymphoma in asymptomatic carriers. *International Journal of Cancer*.

[30] Kubota R, Kawanishi T, Matsubara H, Manns A, Jacobson S (2000). HTLV-I specific IFN-*γ*+ CD8^+^ lymphocytes correlate with the proviral load in peripheral blood of infected individuals. *Journal of Neuroimmunology*.

[31] Kubota R, Nagai M, Kawanishi T, Osame M, Jacobson S (2000). Increased HTLV type 1 tax-specific CD8^+^ cells in HTLV type 1-associated myelopathy/tropical spastic paraparesis: correlation with HTLV type 1 proviral load. *AIDS Research and Human Retroviruses*.

[32] Wodarz D, Hall SE, Usuku K (2001). Cytotoxic T-cell abundance and virus load in human immunodeficiency virus type 1 and human T-cell leukaemia virus type 1. *Proceedings of the Royal Society B*.

[33] Bangham CR, Meekings K, Toulza F (2009). The immune control of HTLV-1 infection: selection forces and dynamics. *Frontiers in Bioscience*.

[34] Asquith B, Mosley AJ, Barfield A (2005). A functional CD8^+^ cell assay reveals individual variation in CD8^+^ cell antiviral efficacy and explains differences in human T-lymphotropic virus type 1 proviral load. *Journal of General Virology*.

[35] Kattan T, MacNamara A, Rowan AG (2009). The avidity and lytic efficiency of the CTL response to HTLV-11. *Journal of Immunology*.

[36] Asquith B, Zhang Y, Mosley AJ (2007). *In vivo* T lymphocyte dynamics in humans and the impact of human T-lymphotropic virus 1 infection. *Proceedings of the National Academy of Sciences of the United States of America*.

[37] Jeffery KJM, Usuku K, Hall SE (1999). HLA alleles determine human T-lymphotropic virus-I (HTLV-I) proviral load and the risk of HTLV-I-associated myelopathy. *Proceedings of the National Academy of Sciences of the United States of America*.

[38] Bieganowska K, Höllsberg P, Buckle GJ (1999). Direct analysis of viral-specific CD8^+^ T cells with soluble HLA- A2/Tax11-19 tetramer complexes in patients with human T cell lymphotropic virus-associated myelopathy. *Journal of Immunology*.

[39] Yashiki S, Fujiyoshi T, Arima N (2001). HLA-A^*^26, HLA-B*4002, HLA-B*4006, and HLA-B*4801 alleles predispose to adult T cell leukemia: the limited recognition of HTLV type 1 tax peptide anchor motifs and epitopes to generate anti-HTLV type 1 tax CD8^+^ cytotoxic T lymphocytes. *AIDS Research and Human Retroviruses*.

[40] Gaudray G, Gachon F, Basbous J, Biard-Piechaczyk M, Devaux C, Mesnard JM (2002). The complementary strand of the human T-cell leukemia virus type 1 RNA genome encodes a bZIP transcription factor that down-regulates viral transcription. *Journal of Virology*.

[41] MacNamara A, Kadolsky U, Bangham CRM, Asquith B (2009). T-cell epitope prediction: rescaling can mask biological variation between MHC molecules. *PLoS Computational Biology*.

[42] MacNamara A, Rowan A, Hilburn S (2010). HLA class I binding of HBZ determines outcome in HTLV-1 infection. *PLoS Pathogens*.

[43] Hilburn S, Rowan A, Demontis M-A (2011). *In vivo* expression of human T-lymphotropic virus type 1 basic leucine-zipper protein generates specific CD8^+^ and CD4^+^ T-lymphocyte responses that correlate with clinical outcome. *Journal of Infectious Diseases*.

[44] Kurihara K, Harashima N, Hanabuchi S (2005). Potential immunogenicity of adult T cell leukemia cells *in vivo*. *International Journal of Cancer*.

[45] Kinoshita T, Shimoyama M, Tobinai K (1989). Detection of mRNA for the tax1/rex1 gene of human T-cell leukemia virus type I in fresh peripheral blood mononuclear cells of adult T-cell leukemia patients and viral carriers by using the polymerase chain reaction. *Proceedings of the National Academy of Sciences of the United States of America*.

[46] Takeda S, Maeda M, Morikawa S (2004). Genetic and epigenetic inactivation of TAX gene in adult t-cell leukemia cells. *International Journal of Cancer*.

[47] Ohashi T, Hanabuchi S, Kato H (2000). Prevention of adult T-cell leukemia-like lymphoproliferative disease in rats by adoptively transferred T cells from a donor immunized with human T-cell leukemia virus type 1 Tax-coding DNA vaccine. *Journal of Virology*.

[48] Hanabuchi S, Ohashi T, Koya Y (2001). Regression of human T-cell leukemia virus type I (HTLV-I)-associated lymphomas in a rat model: peptide-induced T-cell immunity. *Journal of the National Cancer Institute*.

[49] Harashima N, Kurihara K, Utsunomiya A (2004). Graft-versus-Tax Response in adult T-cell leukemia patients after hematopoietic stem cell transplantation. *Cancer Research*.

[50] Harashima N, Tanosaki R, Shimizu Y (2005). Identification of two new HLA-A*1101-restricted tax epitopes recognized by cytotoxic T lymphocytes in an adult T-cell leukemia patient after hematopoietic stem cell transplantation. *Journal of Virology*.

[51] Saito M, Matsuzaki T, Satou Y (2009). *In vivo* expression of the HBZ gene of HTLV-1 correlates with proviral load, inflammatory markers and disease severity in HTLV-1 associated myelopathy/tropical spastic paraparesis (HAM/TSP). *Retrovirology*.

[52] Satou Y, Yasunaga JI, Yoshida M, Matsuoka M (2006). HTLV-I basic leucine zipper factor gene mRNA supports proliferation of adult T cell leukemia cells. *Proceedings of the National Academy of Sciences of the United States of America*.

[53] Suemori K, Fujiwara H, Ochi T (2009). HBZ is an immunogenic protein, but not a target antigen for human T-cell leukemia virus type 1-specific cytotoxic T lymphocytes. *Journal of General Virology*.

[54] Rende F, Cavallari I, Corradin A (2011). Kinetics and intracellular compartmentalization of HTLV-1 gene expression: nuclear retention of HBZ mRNAs. *Blood*.

[55] Matsuoka M, Jeang KT (2010). Human T-cell leukemia virus type 1 (HTLV-1) and leukemic transformation: viral infectivity, Tax, HBZ and therapy. *Oncogene*.

[56] Satou Y, Yasunaga J-I, Zhao T (2011). HTLV-1 bZIP factor induces T-cell lymphoma and systemic inflammation *in vivo*. *PLoS Pathogens*.

[57] Sundaram R, Lynch MP, Rawale S (2004). Protective efficacy of multiepitope human leukocyte antigen-A*0201 restricted cytotoxic T-lymphocyte peptide construct against challenge with human T-cell lymphotropic virus type 1 Tax recombinant vaccinia virus. *Journal of Acquired Immune Deficiency Syndromes*.

[58] Sundaram R, Sun Y, Walker CM, Lemonnier FA, Jacobson S, Kaumaya PTP (2003). A novel multivalent human CTL peptide construct elicits robust cellular immune responses in HLA-A^*^0201 transgenic mice: implications for HTLV-1 vaccine design. *Vaccine*.

[59] Taniguchi Y, Nosaka K, Yasunaga JI (2005). Silencing of human T-cell leukemia virus type I gene transcription by epigenetic mechanisms. *Retrovirology*.

[60] Koiwa T, Hamano-Usami A, Ishida T (2002). 5'-long terminal repeat-selective CpG methylation of latent human T-cell leukemia virus type 1 provirus *in vitro* and *in vivo*. *Journal of Virology*.

